# Automated Compactness Quantitative Metrics for Wrist Bone on Conventional Radiography in Rheumatoid Arthritis: A Clinical Evaluation Study

**DOI:** 10.3390/jimaging12020087

**Published:** 2026-02-18

**Authors:** Jiajing Zhou, Junmu Peng, Haolin Wang, Hiroshi Kataoka, Masaya Mukai, Tunlada Wiriyanukhroh, Tamotsu Kamishima

**Affiliations:** 1 Faculty/Graduate School of Health Sciences, Hokkaido University, Sapporo 060-0812, Hokkaido, Japan; zzzjjj99@163.com (J.Z.); junmupeng@kamiken.net (J.P.); haolin.wang.k3@elms.hokudai.ac.jp (H.W.); tunlada.wi@gmail.com (T.W.); 2 Sapporo City General Hospital, Sapporo 060-8604, Hokkaido, Japan; hiroshi.kataoka@doc.city.sapporo.jp; 3 Soen Chuo Hospital, Sapporo 621-1023, Hokkaido, Japan; mmukai@cocoa.ocn.ne.jp; 4 Phramongkutklao Hospital and College of Medicine, Bangkok 10400, Thailand

**Keywords:** rheumatoid arthritis, computer-aided diagnosis, deep learning, conventional radiography

## Abstract

Rheumatoid arthritis (RA) frequently affects the joints of the hands, with joint space narrowing (JSN) representing an important early marker of structural damage. The semi-quantitative Sharp/van der Heijde (SvdH) scoring system is widely used in clinical practice but is inherently subjective and susceptible to observer variability. Moreover, the complex anatomy of the wrist and substantial overlap of carpal bones pose challenges for automated quantitative assessment of wrist JSN on routine radiographs. This study aimed to introduce a novel quantitative assessment perspective and to clinically validate an automated, compactness-related quantification framework for evaluating wrist JSN in RA. This study initially enrolled 51 patients with RA. After excluding one case with severe carpal fusion that precluded anatomical differentiation, 50 patients (44 females and 6 males) were included in the final analysis. The cohort had a mean age of 61 years (range: 21–82), a median symptom duration of 9 years (IQR: 1–32), and a median follow-up interval for bilateral hand radiographs of 1.06 years (IQR: 0.82–1.30). To quantify global wrist JSN, 10 compactness-related metrics were computed based on the spatial distribution of bone centroids extracted from carpal segmentation masks. These metrics were validated against the wrist JSN subscore of the SvdH score (SvdH-JSN_wrist) and the total Sharp score (TSS) as gold standards. Several distance-based metrics among the compactness-related metrics showed significant negative correlations with the wrist joint space narrowing subscore of the Sharp/van der Heijde score (SvdH-JSN_wrist). Specifically, mean-pairwise-distance (MPD), root-mean-square-radius (RMSR), and median-radius (R50) showed moderate to strong correlations (r = −0.52 to −0.63, all p≤0.0001) that were consistent at BL and FU. Correlations with TSS were weaker overall, with only R50 and its normalized form showing stable negative correlations (r = −0.40 to −0.43, *p* < 0.01). Longitudinal analyses showed limited correlations between metric changes and clinical score changes. The proposed automated compactness quantification framework enables objective and reliable assessment of wrist JSN on standard radiographs and complements conventional scoring systems by supporting automated and standardized evaluation of RA-related wrist structural changes.

## 1. Introduction

Rheumatoid arthritis (RA) is a chronic autoimmune disease characterized by synovial inflammation and clinically manifested as symmetrical polyarthritis [1]. It predominantly affects small joints, particularly those of the hands and wrists [2]. As the disease progresses, structural damage such as bone erosion (BE) and joint space narrowing (JSN) develops, eventually leading to joint deformity and functional impairment in severe cases [3,4]. RA substantially compromises quality of life and imposes a significant socioeconomic burden, with a reported global prevalence of approximately 0.5–1% [2,5,6]. Early detection, accurate diagnosis, and longitudinal monitoring are therefore essential to prevent irreversible joint damage and disability.

The wrist joint is among the earliest and most frequently affected anatomical sites in RA [7]. Radiographic findings such as JSN and BE reflect early structural progression and remain central to disease assessment [8]. Conventional radiography continues to be the most widely used imaging modality in RA because of its low cost, broad availability, and standardized role in diagnosis, staging, and long-term follow-up [9,10]. However, quantitative assessment of wrist JSN on radiographs is particularly challenging. The wrist consists of multiple small carpal bones with complex morphology and close spatial relationships, and radiographs provide only two-dimensional projections. As a result, manual evaluation is highly susceptible to projection variability, bone overlap, and reader subjectivity [11,12]. The Sharp/van der Heijde (SvdH) scoring system is currently the most widely accepted radiographic scoring method for RA [10,13]. Its total score (TSS) integrates JSN and BE in the hands and feet, reflecting cumulative structural damage and long-term prognosis [14,15,16]. Despite its clinical utility, SvdH scoring is semi-quantitative, labor-intensive, and subject to inter- and intra-observer variability [10,17]. Although direct manual measurements can provide quantitative information, they are often impractical in routine workflows and insufficiently sensitive to subtle, continuous morphological changes, particularly within the anatomically complex wrist joint. Consequently, there is a clear need for automated quantitative approaches that can objectively characterize wrist structural changes while maintaining clinical relevance.

In this context, recent studies have increasingly emphasized the importance of explainable artificial intelligence (XAI) and imaging biomarkers in medical image analysis. Imaging biomarkers are defined as quantitative image-derived features with explicit morphological or geometric meaning, enabling transparent and clinically interpretable characterization of disease-related structural changes. Compared with purely data-driven deep learning representations, handcrafted features grounded in predefined anatomical or geometric formulations have been highlighted as inherently explainable and more closely aligned with clinical reasoning [18]. Furthermore, evidence-based XAI frameworks stress that interpretability and empirical clinical relevance should be integral components of quantitative imaging methodologies, particularly in radiological decision support applications [19]. These considerations are especially pertinent in musculoskeletal radiography of peripheral joints, where anatomically interpretable measurements are essential for clinical acceptance and longitudinal assessment.

Recent advances in artificial intelligence have enabled automated joint segmentation and quantitative analysis using radiographs. However, most existing studies have focused on finger joints, where anatomical structures are relatively well separated [20,21]. Automated quantitative assessment of the wrist remains limited due to severe bone overlap, complex joint geometry, and projection-related variability inherent to two-dimensional imaging [22]. Under these conditions, achieving robust and reliable quantitative measurements across different imaging positions remains challenging [23]. To address these limitations, we propose a compactness-based morphological analysis framework for automated assessment of wrist JSN in RA. Rather than relying on local joint space measurements, the proposed approach characterizes the global spatial distribution of carpal bones using centroid-based compactness metrics derived from automated segmentation. The main contributions of this study are as follows:

- **A compactness-based quantitative perspective**: We introduce a global geometric characterization of wrist JSN based on compactness, shifting from conventional local distance measurements to an integrated assessment of carpal bone spatial configuration.
- **Centroid-based compactness metrics**: Using automated carpal bone segmentation with the AP-DPM framework, centroid coordinates of individual carpal bones are extracted to construct a series of compactness-related morphological metrics that are robust to bone overlap and projection variability.
- **Clinical validation against established scoring systems**: The proposed metrics are validated by analyzing their associations with the wrist JSN subscore and total Sharp/van der Heijde score, to evaluate their ability to capture both local wrist structural changes and overall disease severity.

## 2. Materials and Methods

### 2.1. Patients

**Figure 1:**
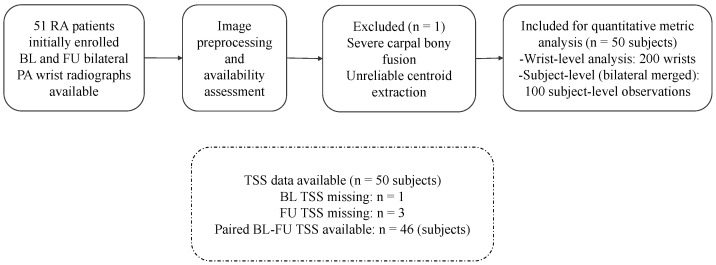
Flowchart of patient inclusion and data availability. 51 patients with rheumatoid arthritis (RA) underwent bilateral posteroanterior wrist radiography at basline (BL) and follow-up (FU). 1 subject was excluded due to severe carpal bony fusion, leaving 50 patients for quantitative analysis. The wrist joint space narrowing subscore of the Sharp/van der Heijde score (JSNS) was analyzed at the wrist (200 wrists) and subject levels (100 observations), and paired BL–FU TSS data were available for 46 subjects.

This study initially enrolled 51 patients with RA (45 females and 6 males). All patients underwent bilateral posteroanterior (PA) conventional radiography examinations at basline (BL) and follow-up (FU). Patient demographics are detailed in Table 1. During data preprocessing, one patient was excluded based on pre-defined image usability criteria. Specifically, this case exhibited severe structural joint destruction, including extensive bony fusion and advanced bone erosion of the carpal joints, which precluded reliable anatomical differentiation of individual carpal bones on the PA radiographic view. Under such conditions, centroid localization and subsequent compactness-related metric computation were deemed unreliable. Accordingly, cases with severe carpal fusion or late-stage structural deformity were considered an intrinsic limitation of the proposed centroid-based compactness analysis framework and were excluded from quantitative analysis. Ultimately, 50 patients were included in the subsequent compactness-related quantitative metric calculations. For JSNS, all 50 included cases had complete BL and FU data. TSS data were derived from the same cohort of subjects. However, due to missing raw scoring data, one patient lacked BL scores, and three lacked FU scores. The patient inclusion process and data availability are summarized in Figure 1.

**Figure 2:**
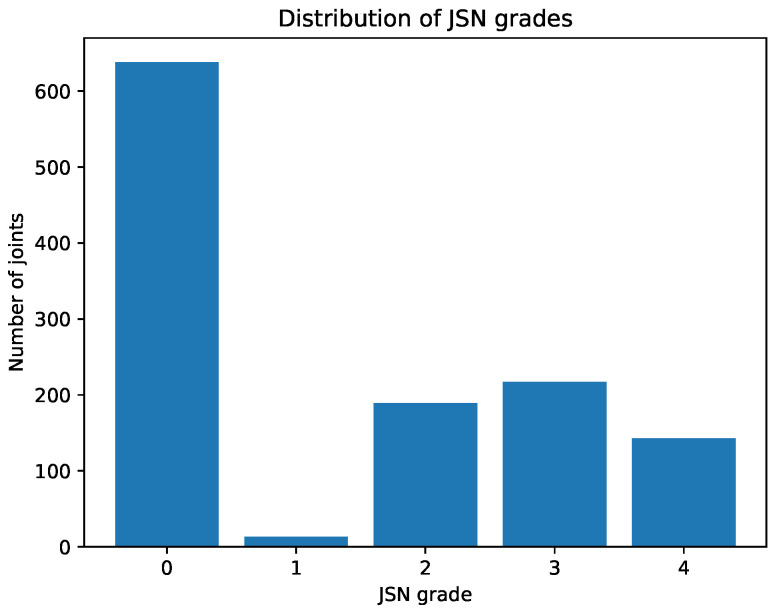
Distribution of joint-level joint space narrowing (JSN) grades (0–4) across all evaluated wrist joints. The histogram summarizes the frequency of individual JSN grades underlying the summed JSN scores used in the correlation analyses.

Following the description of patient characteristics, the severity of structural joint damage in the study cohort was further characterized. The distribution of joint-level wrist JSN grades across all evaluated joints is shown in Figure 2. While a substantial proportion of joints exhibited no narrowing, moderate to severe JSN grades were also frequently observed, indicating marked heterogeneity in wrist structural involvement.

In addition, TSS demonstrated substantial inter-patient variability, with a mean ± standard deviation of 98.6 ± 97.8.

### 2.2. Radiograph Acquisition

All BL and FU radiographs were obtained using the same conventional radiography system (Toshiba KXO-50G, Tokyo, Japan) in standardized acquisition settings. The imaging was performed without an aluminum filter, with a film–focus distance of 1 m, a tube voltage of 45 kV, a tube current of 250 mA and an exposure time of 0.014 s. Subsequently, all images were digitized and stored in Digital Imaging and Communications in Medicine (DICOM) format, with a pixel resolution of 0.15 × 0.15 mm and a 10-bit grayscale depth.

### 2.3. Wrist Sharp/Van Der Heijde JSN Scores

JSN progression was independently evaluated using SvdH scoring method by two experienced radiologists. Their consensus scores were used as the reference standard for subsequent analyzes in this study. JSN was graded for the wrist joints on a five-point scale as follows: grade 0, normal joint space; grade 1, focal narrowing or questionable change; grade 2, generalized narrowing with more than 50% of the original joint space remaining; grade 3, generalized narrowing with less than 50% of the original joint space remaining or presence of subluxation; and grade 4, bony ankylosis or complete joint luxation [10].

### 2.4. Total Sharp/Van Der Heijde Scores

TSS were assessed using the SvdH scoring method by two experienced rheumatologists from the affiliated hospitals. Their consensus scores were used as the reference standard for subsequent analyses in this study. The TSS was calculated as the sum of JSN and BE scores across all evaluated joints, including the hands and wrists, and the feet when available, thereby reflecting the overall extent of structural joint damage.

### 2.5. Segmentation Network and Quantitative Metrics

**Figure 3:**
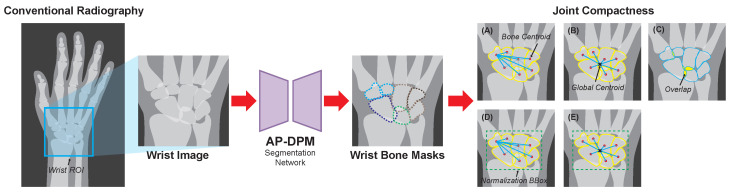
Overview of AP-DPM segmentation network and segmentation masks based wrist JSN quantitative metric. (**A**) illustrates metrics based on local centroid relationships, (**B**) illustrates metrics based on local-global centroids relationships, (**C**) illustrates metrics based on bone overlap size relationships, (**D**,**E**) presents the normalized-BBox versions of (**A**,**B**).

As shown in Figure 3, our proposed quantification framework consists of a wrist-specific segmentation network and a set of wrist bone compactness metrics.

#### 2.5.1. AP-DPM Segmentation Network

For segmentation, we performed AP-DPM [24], a model tailored to the anatomical characteristics of the wrist bones. AP-DPM employs a dual-path architecture combined with adversarial anatomical priors to improve delineation in severely overlapped regions. The network separately predicts the global bone structure and the overlapping areas, which are subsequently refined through a lightweight merging module, enabling enhanced sensitivity to blurred boundaries and joint spaces. In parallel, a discriminator imposes anatomical plausibility by penalizing implausible bone shapes, thereby enforcing structurally realistic predictions. A two-stage training strategy further stabilizes learning and balances segmentation accuracy with anatomical consistency. This design yields contour-precise and occlusion-robust segmentation masks, which serve as reliable inputs for downstream compactness metric computation.

The Quantitative results of AP-DPM consistently demonstrate the superiority of AP-DPM over existing state-of-the-art segmentation approaches across multiple evaluation metrics [24]. In terms of overall region accuracy, AP-DPM achieves the highest Dice score of 0.9784 ± 0.0052, outperforming strong baselines such as nnUNet (0.9765 ± 0.0058) and UNet (0.9231 ± 0.001). More notably, its advantage becomes more pronounced on the boundary-sensitive normalized surface Dice (NSD), where AP-DPM reaches 0.7830 ± 0.0788, compared with 0.7612 ± 0.0572 for nnUNet and 0.7087 ± 0.0784 for UNet. This improvement underscores its enhanced capability to preserve fine anatomical interfaces under severe projection overlap. Consistently lower volumetric overlap error (VOE: 0.0142 ± 0.0096) and relative absolute volume difference (RAVD: 0.0090 ± 0.0021) further confirm the geometric stability and robustness of the proposed framework.

#### 2.5.2. Carpal Compactness Quantitative Metrics

To quantify the spatial compactness and overall geometric arrangement of the seven carpal bones, we purposed a set of metrics based on pairwise mask overlap and centroid distributions, as shown in Table 2. All carpal masks were first resampled to a uniform resolution of 600×600, and the centroid of each bone was extracted to form a compact set of quantitative metrics. These metrics can be grouped into three categories: overlap size-based metrics derived from pairwise overlap size, local centroid–based metrics capturing inter-centroid relationships, and local–global centroid–based metrics characterizing the spatial distribution of centroids with respect to the global centroid, as detailed below.

##### Overlap Size Based Metrics

The degree of anatomical crowding between any two carpals was quantified using the binary overlap of their segmentation masks. For carpal masks $M_{i}$ and $M_{j}$, the overlap size was defined as Equation (1)

(1) $$S_{ij}=\left|\{\;x|M_{i}\left(x\right)=1\wedge M_{j}\left(x\right)=1\;\}\right|,$$

where |·| denotes the number of pixels. Based on all unordered carpal pairs (i,j) with i≠j, the overlap size sum (OSS) and the overlap size standard deviation (OSSD) were computed as Equation (2)

(2) $$OSS=\sum_{i\neq j}S_{ij},\;OSSD=\sqrt{\mathrm{Var}\left(S_{ij}\right)}.$$

##### Local Centroid Based Metrics

To quantify how tightly the carpal centroids cluster around their mean location, we computed the root-mean-square (RMS) radius. Let $c_{k}\in R^{2}$ denote the centroid of the *k*-th carpal bone and μ the global centroid defined as Equation (3). Thus, the RMS radius (RMSR) was then defined as Equation (4)

(3) $$\mu =\frac{1}{n}\sum_{k=1}^{n}c_{k}.$$

(4) $$RMSR=\sqrt{\frac{1}{n}\sum_{k=1}^{n}{∥c_{k}-\mu ∥}^{2}}.$$

Inter-bone spacing was further characterized using the mean pairwise Euclidean distance (MPD) between all carpal centroids, defined as Equation (5)

(5) $$MPD=\frac{1}{n(n-1)}\sum_{\begin{array}{c} i,j=1\\ i\neq j \end{array}}^{n}∥c_{i}-c_{j}∥.$$

##### Local–Global Centroid Based Metrics

To reduce sensitivity to outliers and asymmetric bone arrangements, robust radius measures were derived from the distribution of radial distances to the global centroid. The robust radius was defined as Equation (6).

(6) $$R50=\mathrm{quantile}\left(∥c_{k}-\mu ∥,\;0.5\right),\;R90=\mathrm{quantile}\left(∥c_{k}-\mu ∥,\;0.9\right)$$

**Scale Normalization.** To achieve scale invariance across subjects, all centroid-based compactness metrics were normalized by the diagonal length of the bounding box enclosing all centroids. The bounding box diagonal was computed as Equation (7).

(7) $$L_{\mathrm{bbox}}=\sqrt{{\left(x_{\max}-x_{\min}\right)}^{2}+{\left(y_{\max}-y_{\min}\right)}^{2}},$$

where $\left(x_{\min},y_{\min}\right)$ and $\left(x_{\max},y_{\max}\right)$ denote the minimum and maximum centroid coordinates. The normalized metrics were then defined as Equation (8).

(8) $$RMSR_{n}=\frac{RMSR}{L_{\mathrm{bbox}}},\;MPD_{n}=\frac{MPD}{L_{\mathrm{bbox}}},\;R50_{n}=\frac{R50}{L_{\mathrm{bbox}}},\;R90_{n}=\frac{R90}{L_{\mathrm{bbox}}}.$$

## 3. Results

### 3.1. Comparison Results with JSN

#### 3.1.1. Entire Cohort

Based on the entire cohort of 50 patients, a total of 200 wrist radiographs (50 subjects × bilateral wrists × BL and FU) with available JSN data were included in the correlation analyses. Overall, several centroid-based compactness metrics demonstrated moderate to relatively strong negative correlations with JSN (Table 3).

Among all evaluated compactness-related metrics, the centroid-based distance metrics derived from centroid spatial distribution showed the strongest correlations with JSN. Specifically, RMSR (r=−0.45, p<0.0001), MPD (r=−0.49, p<0.0001) and R50 (r=−0.49, p<0.0001) showing moderate to strong negative correlations with JSN. In contrast, R90 showed a weaker but still statistically significant negative correlation (r=−0.33, p<0.0001).

Among the normalized centroid-based compactness metrics, significant negative correlations with JSN were consistently observed. Both $MPD_{n}$ and $RMSR_{n}$ exhibited moderate correlations (r=−0.45 and r=−0.31, respectively; both p<0.0001), while R50n showed a statistically significant but slightly weaker correlation (r=−0.30, p<0.0001). Notably, R90,n demonstrated a weak positive correlation with JSN (r=0.16, p=0.027).

In contrast, the overlap size–based metrics (OSS and OSSD) showed no statistically significant correlations with JSN in the overall dataset (p>0.05).

The complete correlation coefficients, confidence intervals, and significance levels are presented in Table 3. The complete results for all evaluated metrics are provided in Supplementary Figure S1.

#### 3.1.2. Comparison of Baseline and Follow-Up Results for the Left and Right Hands

In BL radiographs (n = 100) (50 subjects × BL and FU), among the centroid-based compactness metrics, strong and statistically significant negative correlations with JSN were observed. Specifically, RMSR (r=−0.47, p<0.0001), MPD (r=−0.51, p<0.0001), and R50 (r=−0.48, p<0.0001) exhibited high and consistent correlations, all reaching statistical significance. R90 also demonstrated a statistically significant but comparatively weaker negative correlation (r=−0.34, p=0.0006). Among the normalized centroid-based compactness metrics, statistically significant negative correlations with JSN were observed for $RMSR_{n}$ (r=−0.28, p=0.0046), $MPD_{n}$ (r=−0.41, p<0.0001), and R50n (r=−0.28, p=0.0042). In contrast, R90,n did not show a statistically significant correlation with JSN at BL (p>0.05). In addition, the overlap size–based metrics (OSS and OSSD) did not demonstrate statistically significant correlations with JSN at BL (p>0.05).

In FU radiographs (n = 100), similar but slightly strengthened correlation patterns were observed. Among the centroid-based compactness metrics, RMSR (r=−0.44, p<0.0001), MPD (r=−0.47, p<0.0001), and R50 (r=−0.49, p<0.0001) exhibited moderate to strong negative correlations with JSN. R90 demonstrated a weaker but still statistically significant correlation (r=−0.32, p=0.0014). Among the normalized centroid-based compactness metrics, $MPD_{n}$ showed the strongest correlation with JSN (r=−0.48, p<0.0001), followed by $RMSR_{n}$ (r=−0.33, p=0.0008) and R50n (r=−0.31, p=0.0014), all reaching statistical significance. In contrast, R90,n did not demonstrate a significant correlation with JSN at FU (p>0.05). Similarly, the overlap size–based metrics (OSS and OSSD) did not show statistically significant correlations with JSN in FU radiographs (p>0.05).

In the longitudinal change analysis (Δ, n = 50), only the overlap size–based metric ΔOSS exhibited a statistically significant negative correlation with ΔJSN (r=−0.30, p=0.0364). ΔOSSD showed a non-significant trend toward a negative correlation (p>0.05).

**Figure 4:**
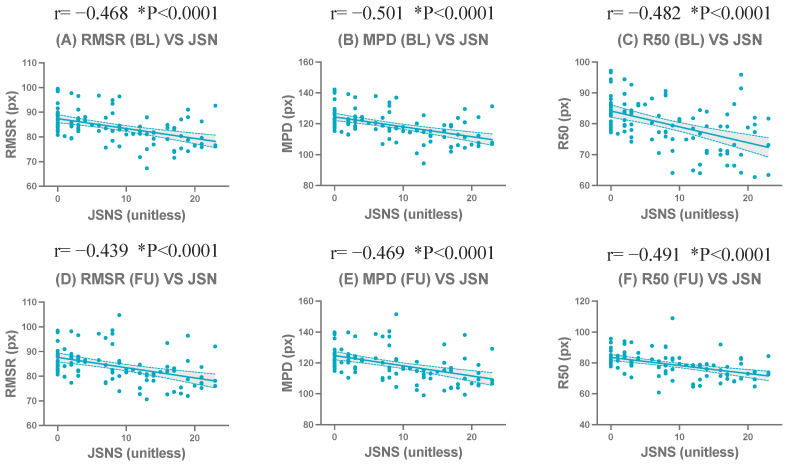
Correlations between the wrist joint space narrowing subscore of the Sharp/van der Heijde score (JSNS) and compactness-related metrics at baseline (BL) and follow-up (FU). Scatter plots show representative metrics: RMSR (**A**,**D**), MPD (**B**,**E**), and R50 (**C**,**F**). The upper row (**A**–**C**) shows BL results, and the lower row (**D**–**F**) shows FU results. * indicates statistical significance (*p* < 0.05).

The complete correlation coefficients, confidence intervals, and significance levels are presented in Table 4 and Table 5. Representative scatter plots of the strongest correlated metrics demonstrated a clear linear negative correlation (Figure 4). Detailed scatter plots for all evaluated metrics at BL and FU are shown in Supplementary Figures S2 and S3.

#### 3.1.3. Comparison of Bilateral Total Scores at Baseline and Follow-Up

Bilateral total JSNS was calculated by summing left and right wrist subscores for each subject at each time point. Thus, 50 subjects were included in the BL and FU analyses, respectively.

In BL radiographs (n = 50), among the centroid-based compactness metrics, strong and statistically significant negative correlations with JSN were observed. Specifically, RMSR (r=−0.55, p<0.0001), MPD (r=−0.59, p<0.0001), and R50 (r=−0.53, p<0.0001) exhibited high and consistent correlations, all reaching statistical significance. R90 also demonstrated a statistically significant but comparatively weaker negative correlation (r=−0.40, p=0.0043). Among the normalized centroid-based compactness metrics, statistically significant negative correlations with JSN were observed for $RMSR_{n}$ (r=−0.28, p=0.0486) and $MPD_{n}$ (r=−0.43, p=0.0018). In contrast, $R50_{n}$ (p=0.0577) and $R90_{n}$ (p>0.05) did not show statistically significant correlations with JSN at BL. In addition, the overlap size–based metrics (OSS and OSSD) did not demonstrate statistically significant correlations with JSN at BL (p>0.05).

In FU radiographs (n = 50), a similar but slightly strengthened correlation pattern was observed. Among the centroid-based compactness metrics, RMSR (r=−0.52, p=0.0001), MPD (r=−0.55, p<0.0001), and R50 (r=−0.58, p<0.0001) exhibited moderate to strong negative correlations with JSN. R90 demonstrated a weaker but still statistically significant correlation (r=−0.39, p=0.0047). Among the normalized centroid-based compactness metrics, $MPD_{n}$ showed the strongest correlation with JSN (r=−0.56, p<0.0001), followed by $RMSR_{n}$ (r=−0.41, p=0.0028) and $R50_{n}$ (r=−0.38, p=0.0072), all reaching statistical significance. In contrast, $R90_{n}$ did not demonstrate a significant correlation with JSN at FU (p>0.05). Similarly, the overlap size–based metrics (OSS and OSSD) did not show statistically significant correlations with JSN in FU radiographs (p>0.05).

**Figure 5:**
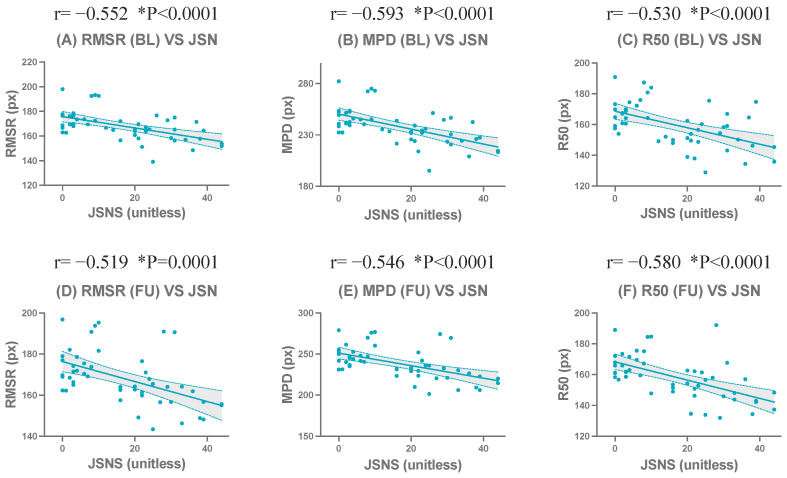
Correlations between centroid-based compactness metrics and the bilateral summed wrist joint space narrowing subscore of the Sharp/van der Heijde score (JSNS) at baseline (BL) and follow-up (FU). Scatter plots show representative metrics: RMSR (**A**,**D**), MPD (**B**,**E**), and R50 (**C**,**F**). The upper row (**A**–**C**) shows BL results, and the lower row (**D**–**F**) shows FU results. * indicates statistical significance (*p* < 0.05).

The complete correlation coefficients, confidence intervals, and significance levels are presented in Table 6. Representative scatter plots of the strongest correlated metrics demonstrated a clear linear negative correlation (Figure 5). Detailed scatter plots for all evaluated metrics at BL and FU are shown in Supplementary Figures S4 and S5.

#### 3.1.4. Comparison of Timepoint-Specific Correlations in the Left and Right Hands

In each subgroup analysis, n = 50 represents the number of wrist radiographs included. Specifically, left and right wrists were analyzed separately, and baseline and follow-up timepoints were evaluated independently, without summation across hands or timepoints.

In the left hand radiographs at BL (n = 50), centroid-based compactness metrics demonstrated strong and statistically significant negative correlations with JSN. Specifically, RMSR (r=−0.56, p<0.0001), MPD (r=−0.61, p<0.0001), and R50 (r=−0.55, p<0.0001) exhibited strong and consistent correlations with JSN. R90 also showed a statistically significant but comparatively weaker negative correlation (r=−0.32, p=0.0235). Among the normalized centroid-based compactness metrics, $MPD_{n}$ (r=−0.53, p<0.0001) exhibited a strong negative correlation with JSN, while $RMSR_{n}$ (r=−0.37, p=0.0082) and $R50_{n}$ (r=−0.33, p=0.0187) also showed statistically significant correlations. In contrast, $R90_{n}$ (r=0.34, p=0.0151) demonstrated a weak positive correlation with JSN.

At FU in the left hand (n = 50), a largely consistent correlation pattern was observed. Strong and statistically significant negative correlations with JSN were again found for R50 (r=−0.57, p<0.0001), MPD (r=−0.55, p<0.0001), and RMSR (r=−0.52, p<0.0001). R90 remained significantly correlated (r=−0.40, p=0.0043). Among the normalized metrics, $MPD_{n}$ continued to demonstrate a strong negative correlation (r=−0.54, p<0.0001), followed by $RMSR_{n}$ (r=−0.41, p=0.0031) and $R50_{n}$ (r=−0.31, p=0.0299), whereas $R90_{n}$ did not reach statistical significance (p>0.05).

In the right hand radiographs at BL (n = 50), a similar correlation pattern was observed, although the overall strength of the correlations was slightly weaker than that of the left hand. Among the centroid-based compactness metrics, RMSR (r=−0.39, p=0.0052), MPD (r=−0.41, p=0.0030), and R50 (r=−0.40, p=0.0036) showed moderate and statistically significant negative correlations with JSN. R90 also demonstrated a statistically significant negative correlation (r=−0.37, p=0.0080). However, among the normalized metrics, none reached statistical significance at BL in the right hand (p>0.05).

At FU in the right hand (n = 50), the correlation strength slightly increased compared with BL. Significant negative correlations with JSN were observed for R50 (r=−0.44, p=0.0015), MPD (r=−0.40, p=0.0038), and RMSR (r=−0.37, p=0.0080). Among the normalized metrics, $MPD_{n}$ (r=−0.41, p=0.0028) and $R50_{n}$ (r=−0.32, p=0.0257) exhibited statistically significant negative correlations with JSN, whereas $RMSR_{n}$ and $R90_{n}$ did not reach statistical significance (p>0.05).

Across all timepoints and both hands, OSS and OSSD consistently showed no statistically significant correlations with JSN (p>0.05).

**Figure 6:**
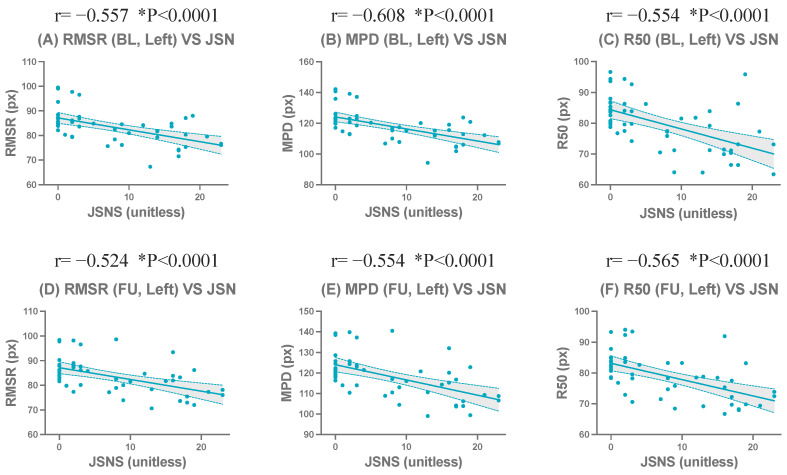
Correlations between centroid-based compactness metrics and the wrist joint space narrowing subscore of the Sharp/van der Heijde score (JSNS) for the left hand at baseline (BL) and follow-up (FU). Scatter plots show representative metrics: RMSR (**A**,**D**), MPD (**B**,**E**), and R50 (**C**,**F**). The upper row (**A**–**C**) shows results for the left hand, and the lower row (**D**–**F**) shows for the right hand. * indicates statistical significance (*p* < 0.05).

**Figure 7:**
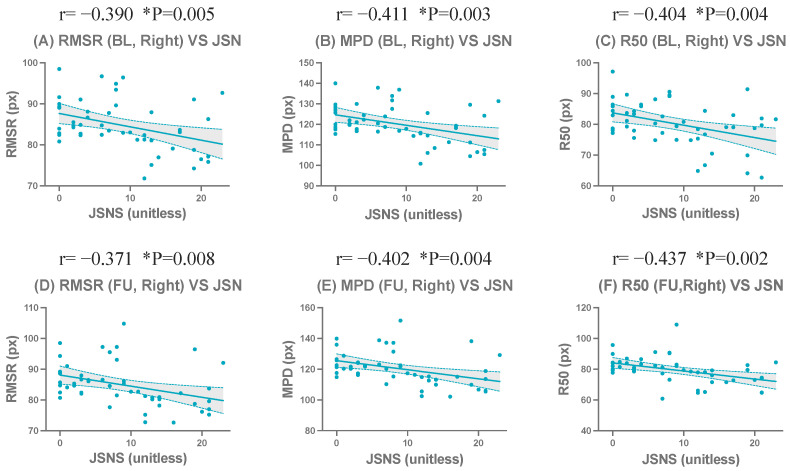
Correlations between centroid-based compactness metrics and the wrist joint space narrowing subscore of the Sharp/van der Heijde score (JSNS) for the Right hand at baseline (BL) and follow-up (FU). Scatter plots show representative metrics: RMSR (**A**,**D**), MPD (**B**,**E**), and R50 (**C**,**F**). The upper row (**A**–**C**) shows results for the left hand, and the lower row (**D**–**F**) shows for the right hand. * indicates statistical significance (*p* < 0.05).

The complete correlation coefficients, confidence intervals, and significance levels are presented in Table 7. Representative scatter plots of the strongest correlated metrics demonstrated a clear linear negative correlation (Figure 6 and Figure 7). Corresponding results for all evaluated metrics in the left and right hands are presented in Supplementary Figures S6–S9.

#### 3.1.5. Comparison of Pooled Correlations in the Left and Right Hands

For hand-specific analyses, the left and right hands were analyzed separately, and BL and FU radiographs were pooled. Accordingly, 50 subjects contributed two time-point observations, yielding 100 left-hand radiographs and 100 right-hand radiographs, respectively.

In the left hand radiographs (n = 100), centroid-based compactness metrics demonstrated moderate to strong and statistically significant negative correlations with JSN. Specifically, RMSR (r=−0.54, p<0.0001), MPD (r=−0.58, p<0.0001), and R50 (r=−0.56, p<0.0001) exhibited strong and consistent correlations with JSN. R90 also showed a statistically significant but comparatively weaker negative correlation (r=−0.36, p=0.0002). Among the normalized centroid-based compactness metrics, significant negative correlations with JSN were observed for $RMSR_{n}$ (r=−0.39, p<0.0001), $MPD_{n}$ (r=−0.53, p<0.0001), and $R50_{n}$ (r=−0.32, p=0.0013). In contrast, $R90_{n}$ demonstrated a weak positive correlation with JSN (r=0.21, p=0.036).

In the right hand radiographs (n = 100), a similar correlation pattern was observed, although the overall strength of the correlations was consistently weaker than that of the left hand. Among the centroid-based compactness metrics, RMSR (r=−0.38, p=0.0001), MPD (r=−0.40, p<0.0001), and R50 (r=−0.42, p<0.0001) showed moderate and statistically significant negative correlations with JSN. R90 demonstrated a weaker but still statistically significant correlation (r=−0.30, p=0.0021). Among the normalized centroid-based compactness metrics, $RMSR_{n}$ (r=−0.21, p=0.0396), $MPD_{n}$ (r=−0.34, p=0.0005), and $R50_{n}$ (r=−0.27, p=0.0074) exhibited statistically significant negative correlations with JSN. In contrast, $R90_{n}$ did not demonstrate a statistically significant correlation with JSN (p>0.05).

For the overlap size–based metrics (OSS and OSSD), no statistically significant correlations with JSN were observed in either the left or right hand (p>0.05).

**Figure 8:**
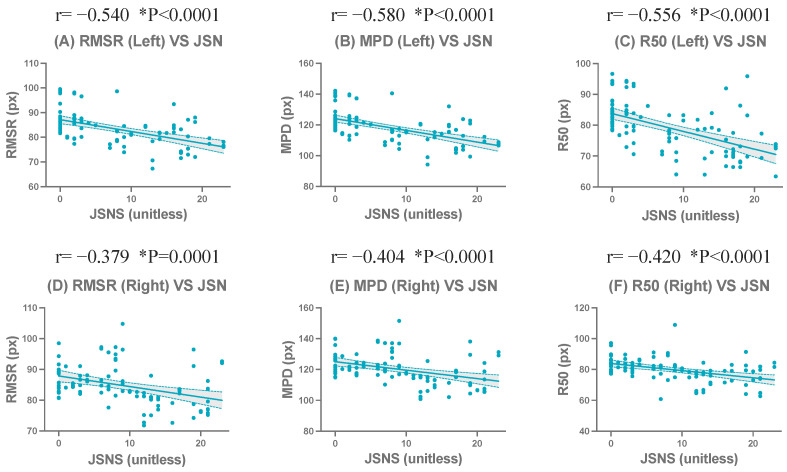
Correlations between centroid-based compactness metrics and the wrist joint space narrowing subscore of the Sharp/van der Heijde score (JSNS) in the left and right hands at baseline (BL) and follow-up (FU). Scatter plots show representative metrics: RMSR (**A**,**D**), MPD (**B**,**E**), and R50 (**C**,**F**). The upper row (**A**–**C**) shows results for the left hand, and the lower row (**D**–**F**) shows for the right hand. * indicates statistical significance (*p* < 0.05).

The complete correlation coefficients, confidence intervals, and significance levels are presented in Table 8. Representative scatter plots of the strongest correlated metrics demonstrated a clear linear negative correlation (Figure 8). Corresponding scatter plots for all evaluated metrics in the left and right hands are shown in Supplementary Figures S10 and S11.

### 3.2. Comparison Results with TSS

#### 3.2.1. Entire Cohort

Based on the final cohort of 50 patients, only patients with paired BL and FU TSS assessments were included in the correlation analyses. As a result, 4 patients (1 missing BL data and 3 missing FU data) were excluded, yielding a total of 46 patients with complete paired TSS data. Overall, several centroid-based compactness metrics demonstrated weak to moderate negative correlations with TSS (Table 9).

Among all evaluated compactness-related metrics, the centroid-based distance metrics derived from centroid spatial distribution exhibited relatively higher correlations with TSS in the overall analysis. R50 consistently showed moderate negative correlations with TSS and reached strong statistical significance (r=−0.43, p<0.0001), whereas MPD demonstrated weaker but statistically significant correlations with TSS (r=−0.30, p=0.0042). RMSR also showed a statistically significant but comparatively weaker correlation with TSS (r=−0.27, p=0.010), while R90 did not reach statistical significance (p>0.05).

Among the normalized centroid-based compactness metrics, stronger correlations with TSS were observed. Both $MPD_{n}$ and $R50_{n}$ demonstrated moderate negative correlations, with correlation coefficients of r=−0.48 and r=−0.47, respectively (both p<0.0001). $RMSR_{n}$ also exhibited a statistically significant correlation with TSS (r=−0.40, p<0.0001), whereas $R_{90,n}$ showed no significant correlation (p>0.05).

In contrast, the overlap size–based metrics (OSS and OSSD) showed no statistically significant correlations with TSS in the overall dataset (p>0.05).

The complete correlation coefficients, confidence intervals, and significance levels are presented in Table 9. The complete results for all evaluated metrics are provided in Supplementary Figure S12.

#### 3.2.2. Comparison of Baseline and Follow-Up

In BL radiographs (n = 46), among the centroid-based compactness metrics, R50 showed a statistically significant negative correlation with TSS (r=−0.41, p=0.0044), while MPD also demonstrated a statistically significant negative correlation (r=−0.31, p=0.0386). In contrast, both RMSR and R90 did not show statistically significant correlations with TSS at BL (p>0.05). Among the normalized centroid-based compactness metrics, $MPD_{n}$ (r=−0.38, p=0.0084) and $R50_{n}$ (r=−0.41, p=0.0051) demonstrated statistically significant negative correlations with TSS, whereas the remaining normalized metrics ($RMSR_{n}$ and $R_{90,n}$), did not reach statistical significance (p>0.05). In addition, the overlap size–based metrics (OSS and OSSD) did not show statistically significant correlations with TSS at BL (p>0.05).

In FU radiographs (46), stronger and more consistent correlations with TSS were observed. Among the centroid-based compactness metrics, R50 showed a statistically significant negative correlation with TSS (r=−0.44, p=0.0023), whereas MPD demonstrated a borderline but non-significant association (r=−0.29, p=0.051). Both RMSR and R90 did not reach statistical significance (p>0.05). Among the normalized centroid-based compactness metrics, strong negative correlations with TSS were observed. $RMSR_{n}$ (r=−0.54, p=0.0001), $MPD_{n}$ (r=−0.59, p<0.0001), and $R50_{n}$ (r=−0.53, p=0.0002) all demonstrated statistically significant negative correlations with TSS. In contrast, $R_{90,n}$ did not show a significant correlation (p>0.05). Similarly, the overlap size–based metrics (OSS and OSSD) did not demonstrate statistically significant correlations with TSS at FU (p>0.05).

In the longitudinal change analysis (Δ), only a limited number of centroid-based compactness metrics, including ΔRMSR, ΔMPD, and ΔR90, exhibited statistically significant negative correlations with ΔTSS (p<0.05), whereas the normalized centroid-based compactness metrics and overlap size–based metrics did not demonstrate significant correlations (p>0.05).

**Figure 9:**
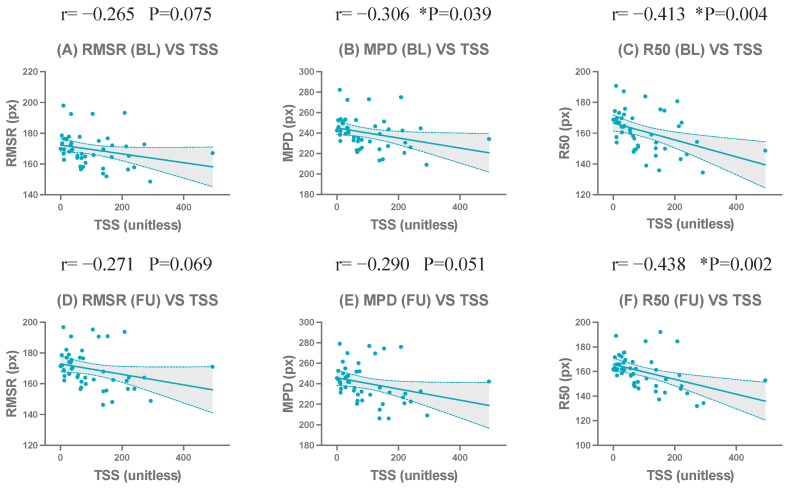
Scatter plots showing correlations between representative compactness-related metrics and the total Sharp/van der Heijde score (TSS) at baseline (BL) and follow-up (FU). Scatter plots show representative metrics: RMSR (**A**,**D**), MPD (**B**,**E**), and R50 (**C**,**F**). The upper row (**A**–**C**) shows BL results, and the lower row (**D**–**F**) shows FU results. * indicates statistical significance (*p* < 0.05).

The complete correlation coefficients, confidence intervals, and significance levels are presented in Table 10 and Table 11. Representative scatter plots of the strongest correlated metrics demonstrated a clear linear negative correlation (Figure 9). Complete correlation results for all metrics with TSS at BL and FU are provided in Supplementary Figures S13 and S14.

## 4. Discussion

### 4.1. Overall Performance of Centroid-Based Compactness Metrics for Wrist JSN Assessment

This study proposes a novel quantitative framework for objectively characterizing wrist joint space narrowing (JSN) in patients with rheumatoid arthritis (RA). Chronic synovitis in RA leads to progressive cartilage loss and gradual joint space narrowing, which underlie the radiographic assessment of structural damage [25,26]. However, most previous studies have relied on traditional semi-quantitative scoring systems, such as the Sharp/van der Heijde (SvdH) method, which depend on manual interpretation, are inherently subjective, and are relatively insensitive to subtle structural changes [27].

Existing automated approaches have mainly focused on local joint boundary detection or overall disease classification, which limits their ability to capture the global spatial contraction of the wrist during disease progression [21,28]. This limitation is particularly relevant for the wrist, where anatomical complexity and overlapping carpal bones reduce the robustness of local joint space measurements on two-dimensional radiographs [29]. To address these challenges, the present study introduces centroid-based compactness metrics derived from the spatial distribution of carpal bone centroids, aiming to characterize the overall contraction trend of the wrist as JSN progresses.

Our results demonstrate that several distance-based centroid metrics showed statistically significant negative correlations with manual JSN scores across the overall cohort and at baseline (BL) and follow-up (FU). Among all evaluated metrics, mean pairwise distance (MPD), root mean square radius (RMSR), and median radius (R50) consistently exhibited the strongest correlations with JSN. These findings indicate that increasing JSN is associated with greater global spatial contraction of carpal bone centroids, supporting the biological plausibility and clinical relevance of the proposed compactness framework.

### 4.2. Sensitivity to Early and Progressive Structural Changes at Baseline and Follow-Up

At baseline, several centroid-based compactness metrics demonstrated significant negative correlations with JSN, suggesting that even subtle early joint space narrowing can influence the global spatial configuration of the carpal bones. This finding indicates that compactness metrics may be sensitive to early structural alterations that are difficult to quantify using conventional radiographic measurements.

At follow-up, the same negative correlation trends were maintained, although longitudinal changes in compactness metrics showed weaker associations with changes in JSN. This observation is likely attributable to the relatively short follow-up interval and the long-standing disease duration in the studied cohort, where substantial structural progression within one year is not always expected. Importantly, the consistency of correlation strength between BL and FU suggests that centroid-based compactness reflects a stable, reproducible imaging feature related to structural narrowing rather than measurement noise.

### 4.3. Bilateral Robustness and Symmetry Considerations

Bilateral analyses showed that centroid-based compactness metrics were consistently negatively correlated with JSN in both wrists. Although slight differences in correlation strength were observed between the left and right hands, these variations may reflect differences in functional loading, positioning during radiographic acquisition, or image quality [23,30]. Overall, the consistent bilateral correlation patterns indicate that the proposed metrics robustly capture JSN-related structural changes across both wrists, supporting their applicability in routine bilateral assessment.

### 4.4. Comparison with TSS and Implications for Assessment Scope

When compared with the total Sharp/van der Heijde score (TSS), centroid-based compactness metrics generally showed weaker correlations than those observed with wrist-specific JSN scores. This difference is expected, as TSS reflects cumulative, multi-joint structural damage represented as JSN and BE in the bilateral hand and foot joints, whereas the proposed compactness metrics focus on local wrist geometry [31]. Structural changes at the wrist do not necessarily progress synchronously with systemic joint damage [32]. Therefore, the relatively weaker association with TSS highlights the complementary nature of the proposed metrics: they are better suited for capturing local wrist-specific structural alterations related to JSN rather than serving as a surrogate for whole-body disease burden.

### 4.5. Advantages over Traditional Scoring Systems and Existing Automated Methods

Traditional radiographic scoring systems remain the clinical reference standard but are time-consuming and limited in their ability to detect subtle or continuous structural changes [33]. Recent deep learning–based methods have improved objectivity by automating joint space width estimation or texture-based assessment [12]; however, these approaches primarily rely on localized joint measurements and remain vulnerable to projection variability and bone overlap in the wrist [34].

In contrast, the proposed compactness framework quantifies the global spatial distribution of multiple carpal bones using centroid-based metrics. By focusing on overall geometric contraction rather than individual joint boundaries, this approach is inherently less sensitive to local segmentation errors and projection-related variability. As demonstrated in this study, centroid-based distance metrics showed stable performance across BL, FU, and bilateral analyses, with correlations that were generally comparable to or superior to those of previously reported computer-aided diagnosis metrics based on local joint characteristics [35].

### 4.6. Biological Interpretation, Limitations, and Future Perspectives

RA is characterized by heterogeneous, sometimes asymmetric, structural progression, particularly in the early stages. Localized and diffuse JSN may coexist, and early changes can be difficult to detect using conventional joint-level scoring alone. By integrating centroid information from multiple carpal bones, the proposed compactness metrics provide a global representation of wrist structural remodeling, capturing an aspect of disease progression that has received limited attention in prior radiographic studies.

Several limitations should be acknowledged. First, the sample size and relatively short follow-up period limit the assessment of sensitivity to short-term progression. Larger cohorts with longer longitudinal follow-up are required to fully evaluate the responsiveness of compactness metrics to disease progression. Second, in advanced RA with severe carpal fusion or marked deformity, reliable segmentation and centroid extraction become infeasible. In such cases, quantitative geometric assessment is subject to ceiling effects and has limited clinical utility. Accordingly, the proposed framework is most suitable for early to moderate RA, where carpal anatomy remains sufficiently distinguishable and quantitative monitoring of structural change is clinically meaningful.

Despite these limitations, this study demonstrates that automated centroid-based compactness metrics can reliably capture global structural changes in the wrist associated with JSN. When used in conjunction with established semi-quantitative scoring systems, this approach provides a complementary, objective tool for standardized radiographic assessment and may facilitate future applications in clinical follow-up and clinical trials.

## 5. Conclusions

This study proposes an automated compactness-related quantitative assessment framework based on AP-DPM for the objective assessment of wrist JSN in patients with RA. The results demonstrate that compactness-related quantitative metrics derived from the spatial distribution of carpal bone centroids can stably capture changes in wrist compactness correlated with JSN and exhibit good measurement stability. In clinical validation, this method shows strong consistency with the JSNS, and comparative analysis with the TSS further clarifies its assessment focus and scope of application. Overall, this automated compactness-related quantitative assessment framework provides a feasible and objective approach for quantifying joint space narrowing–related structural changes in the wrist of patients with RA and lays the foundation for future standardized automated imaging assessments.

## Figures and Tables

**Table 1:** Baseline (BL) clinical and laboratory characteristics of patients with rheumatoid arthritis (RA).

Characteristic	Value
Number of patients	50
Age, years, mean (range)	61 (21–82)
Sex, n (female/male)	44/6
Symptom duration, years, median (IQR)	9 (1–32)
Interval between baseline and follow-up radiographs, years, median (IQR)	1.06 (0.82–1.30)
Structural remission status, n (remission/non-remission)	30/20
ESR, mm/h, median (IQR)	15 (4–76)
CRP, mg/dL, median (IQR)	0.14 (0–6.6)
Swollen joint count, median (IQR)	0 (0–5)
Tender joint count, median (IQR)	0 (0–17)
DAS28-CRP, median (IQR)	1.84 (0.99–3.85)
DAS28-ESR, median (IQR)	2.64 (1.13–4.95)
Total SHS at baseline, median (IQR)	67 (0–494)
Total SHS at follow-up, median (IQR)	67.5 (0–494)
Biologic agents used, n	
None	0
Infliximab	17
Adalimumab	7
Tocilizumab	3
Etanercept	19
Abatacept	4

CRP, C-reactive protein; DAS28, disease activity score in 28 joints; ESR, erythrocyte sedimentation rate; SHS, Sharp–van der Heijde score; IQR, interquartile range.

**Table 2:** Compactness quantitative metrics for carpal bone.

Category	Full Name	Abbreviation
Overlap size based	Overlap size sum	OSS
	Overlap size standard deviation	OSSD
Local centroid based	Root mean square radius	RMSR
	Mean pairwise distance	MPD
local–global centroid based	Median radius	R50
	90th-percentile radius	R90
Local centroid based (normalized)	Normalized RMS radius	$RMSR_{n}$
	Normalized mean pairwise distance	$MPD_{n}$
local–global centroid based (normalized)	Normalized median radius	$R50_{n}$
	Normalized 90th-percentile radius	$R90_{n}$

**Table 3:** Correlations between compactness-related metrics and the wrist joint space narrowing subscore of the Sharp/van der Heijde score (JSNS) (n = 200).

Metric	r	95% CI	*p*
OSS	−0.050	−0.187 to 0.090	0.485
OSSD	−0.058	−0.196 to 0.081	0.412
RMSR	−0.453	−0.556 to −0.335	<0.0001
MPD	−0.485	−0.585 to −0.371	<0.0001
R50	−0.486	−0.586 to −0.373	<0.0001
R90	−0.327	−0.446 to −0.197	<0.0001
RMSR_*n*_	−0.306	−0.427 to −0.175	<0.0001
MPD_*n*_	−0.446	−0.551 to −0.327	<0.0001
R50_*n*_	−0.298	−0.420 to −0.166	<0.0001
R90_*n*_	0.157	0.018 to 0.289	0.0266

**Table 4:** Correlations between the wrist joint space narrowing subscore of the Sharp/van der Heijde score (JSNS) and compactness-related metrics at baseline (BL) and follow-up (FU) (n = 100 per time point).

Metric	Baseline (BL)			Follow-Up (FU)		
	r	95% CI	*p*	r	95% CI	*p*
OSS	−0.017	−0.212 to 0.181	0.871	−0.085	−0.276 to 0.114	0.403
OSSD	−0.004	−0.201 to 0.192	0.966	−0.119	−0.309 to 0.079	0.237
RMSR	−0.468	−0.609 to −0.300	<0.0001	−0.439	−0.585 to −0.266	<0.0001
MPD	−0.505	−0.638 to −0.343	<0.0001	−0.469	−0.609 to −0.300	<0.0001
R50	−0.482	−0.620 to −0.316	<0.0001	−0.491	−0.627 to −0.326	<0.0001
R90	−0.339	−0.502 to −0.153	0.0006	−0.316	−0.483 to −0.128	0.0014
$RMSR_{n}$	−0.281	−0.453 to −0.090	0.0046	−0.331	−0.496 to −0.144	0.0008
$MPD_{n}$	−0.411	−0.562 to −0.233	<0.0001	−0.484	−0.622 to −0.318	<0.0001
$R50_{n}$	−0.284	−0.455 to −0.092	0.0042	−0.315	−0.482 to −0.126	0.0014
$R90_{n}$	0.147	−0.051 to 0.334	0.144	0.166	−0.031 to 0.351	0.098

**Table 5:** Correlations between changes in the wrist joint space narrowing subscore of the Sharp/van der Heijde score (ΔJSNS) and changes in compactness-related metrics (Δmetrics) (n = 50).

ΔMetric	r	95% CI	*p*
Δ OSS	−0.297	−0.531 to −0.020	0.0364
Δ OSSD	−0.221	−0.470 to 0.062	0.1239
Δ RMSR	0.059	−0.223 to 0.332	0.6844
Δ MPD	0.077	−0.206 to 0.348	0.5956
Δ R50	0.079	−0.204 to 0.350	0.5864
Δ R90	−0.012	−0.289 to 0.268	0.9363
Δ $RMSR_{n}$	0.006	−0.273 to 0.284	0.9651
Δ $MPD_{n}$	0.014	−0.265 to 0.291	0.9226
Δ $R50_{n}$	0.010	−0.270 to 0.287	0.9473
Δ $R90_{n}$	−0.127	−0.392 to 0.157	0.3793

**Table 6:** Correlations between the wrist joint space narrowing subscore of the Sharp/van der Heijde score (JSNS) and compactness-related metrics at baseline (BL) and follow-up (FU), based on summed bilateral (left + right) wrist values (n = 50 per time point).

Metric	Baseline (BL)			Follow-Up (FU)		
	r	95% CI	*p*	r	95% CI	*p*
OSS	−0.012	−0.289 to 0.267	0.935	−0.082	−0.352 to 0.201	0.572
OSSD	−0.006	−0.284 to 0.273	0.969	−0.118	−0.383 to 0.166	0.417
RMSR	−0.552	−0.720 to −0.323	<0.0001	−0.519	−0.696 to −0.281	0.0001
MPD	−0.593	−0.748 to −0.377	<0.0001	−0.546	−0.716 to −0.316	<0.0001
R50	−0.530	−0.705 to −0.296	<0.0001	−0.580	−0.739 to −0.360	<0.0001
R90	−0.397	−0.608 to −0.134	0.0043	−0.394	−0.606 to −0.130	0.0047
$RMSR_{n}$	−0.280	−0.518 to −0.002	0.0486	−0.415	−0.621 to −0.154	0.0028
$MPD_{n}$	−0.431	−0.633 to −0.173	0.0018	−0.560	−0.725 to −0.333	<0.0001
$R50_{n}$	−0.270	−0.510 to 0.009	0.0577	−0.376	−0.592 to −0.108	0.0072
$R90_{n}$	0.207	−0.076 to 0.459	0.149	0.170	−0.114 to 0.428	0.237

**Table 7:** Correlations between the wrist joint space narrowing subscore of the Sharp/van der Heijde score (JSNS) and compactness-related metrics at baseline (BL) and follow-up (FU) for the left and right hands (n = 50 per subgroup).

Metric	Left Hand BL			Left Hand FU			Right Hand BL			Right Hand FU		
	r	95% CI	*p*	r	95% CI	*p*	r	95% CI	*p*	r	95% CI	*p*
OSS	0.029	−0.252 to 0.305	0.8425	−0.023	−0.300 to 0.257	0.8735	−0.058	−0.331 to 0.224	0.6903	−0.141	−0.404 to 0.143	0.3279
OSSD	0.047	−0.234 to 0.321	0.7460	−0.089	−0.359 to 0.194	0.5382	−0.058	−0.331 to 0.224	0.6879	−0.156	−0.416 to 0.128	0.2809
RMSR	−0.557	−0.723 to −0.330	<0.0001	−0.524	−0.700 to −0.288	<0.0001	−0.390	−0.603 to −0.125	0.0052	−0.371	−0.589 to −0.104	0.0080
MPD	−0.608	−0.758 to −0.396	<0.0001	−0.554	−0.721 to −0.326	<0.0001	−0.411	−0.619 to −0.150	0.0030	−0.402	−0.612 to −0.139	0.0038
R50	−0.554	−0.721 to −0.326	<0.0001	−0.565	−0.729 to −0.340	<0.0001	−0.404	−0.613 to −0.141	0.0036	−0.437	−0.638 to −0.181	0.0015
R90	−0.320	−0.549 to −0.046	0.0235	−0.397	−0.609 to −0.134	0.0043	−0.371	−0.589 to −0.103	0.0080	−0.239	−0.485 to 0.043	0.0954
$RMSR_{n}$	−0.370	−0.588 to −0.102	0.0082	−0.411	−0.618 to −0.149	0.0031	−0.177	−0.434 to 0.106	0.2180	−0.238	−0.484 to 0.044	0.0966
$MPD_{n}$	−0.526	−0.702 to −0.290	<0.0001	−0.544	−0.714 to −0.313	<0.0001	−0.274	−0.513 to 0.005	0.0541	−0.414	−0.621 to −0.154	0.0028
$R50_{n}$	−0.331	−0.558 to −0.058	0.0187	−0.307	−0.540 to −0.032	0.0299	−0.212	−0.463 to 0.070	0.1386	−0.315	−0.546 to −0.041	0.0257
$R90_{n}$	0.342	0.070 to 0.566	0.0151	0.087	−0.197 to 0.356	0.5497	0.003	−0.276 to 0.281	0.9857	0.245	−0.036 to 0.490	0.0867

**Table 8:** Correlations between the wrist joint space narrowing subscore of the Sharp/van der Heijde score (JSNS) and compactness-related metrics using pooled baseline and follow-up measurements for the left and right hands (BL and FU pooled; n = 100 per hand).

Metric	Left Hand (BL and FU Pooled)			Right Hand (BL and FU Pooled)		
	r	95% CI	*p*	r	95% CI	*p*
OSS	0.004	−0.193 to 0.200	0.9712	−0.099	−0.290 to 0.100	0.3288
OSSD	−0.013	−0.209 to 0.184	0.8979	−0.106	−0.296 to 0.092	0.2935
RMSR	−0.540	−0.666 to −0.384	<0.0001	−0.379	−0.535 to −0.197	0.0001
MPD	−0.580	−0.697 to −0.433	<0.0001	−0.404	−0.557 to −0.226	<0.0001
R50	−0.556	−0.679 to −0.404	<0.0001	−0.420	−0.570 to −0.244	<0.0001
R90	−0.360	−0.520 to −0.176	0.0002	−0.304	−0.472 to −0.114	0.0021
$RMSR_{n}$	−0.389	−0.544 to −0.209	<0.0001	−0.206	−0.387 to −0.010	0.0396
$MPD_{n}$	−0.533	−0.661 to −0.377	<0.0001	−0.341	−0.504 to −0.155	0.0005
$R50_{n}$	−0.318	−0.484 to −0.129	0.0013	−0.267	−0.440 to −0.074	0.0074
$R90_{n}$	0.210	0.0137 to 0.3899	0.0364	0.128	−0.0701 to 0.3165	0.2042

**Table 9:** Correlations between compactness-related metrics and the total Sharp/van der Heijde score (TSS) (n = 92).

Metric	r	95% CI	*p*
OSS	−0.052	−0.255 to 0.154	0.620
OSSD	−0.055	−0.257 to 0.152	0.605
RMSR	−0.267	−0.448 to −0.066	0.010
MPD	−0.296	−0.472 to −0.097	0.0042
R50	−0.425	−0.579 to −0.241	<0.0001
R90	−0.148	−0.342 to 0.059	0.159
RMSR_*n*_	−0.403	−0.561 to −0.216	<0.0001
MPD_*n*_	−0.483	−0.626 to −0.309	<0.0001
R50_*n*_	−0.465	−0.611 to −0.287	<0.0001
R90_*n*_	0.030	−0.176 to 0.233	0.779

**Table 10:** Correlations between compactness-related metrics and the total Sharp/van der Heijde score (TSS) at baseline (BL, n = 46) and follow-up (FU, n = 46).

Metric	Baseline (BL, n = 46)			Follow-Up (FU, n = 46)		
	r	95% CI	*p*	r	95% CI	*p*
OSS	−0.029	−0.317 to 0.263	0.848	−0.076	−0.359 to 0.219	0.614
OSSD	−0.010	−0.300 to 0.281	0.947	−0.103	−0.382 to 0.193	0.494
RMSR	−0.265	−0.516 to 0.027	0.075	−0.271	−0.520 to 0.021	0.069
MPD	−0.306	−0.548 to −0.017	0.039	−0.290	−0.535 to 0.001	0.051
R50	−0.413	−0.628 to −0.139	0.004	−0.438	−0.646 to −0.169	0.002
R90	−0.137	−0.411 to 0.159	0.363	−0.158	−0.429 to 0.139	0.294
$RMSR_{n}$	−0.275	−0.523 to 0.017	0.065	−0.540	−0.718 to −0.296	0.0001
$MPD_{n}$	−0.384	−0.607 to −0.106	0.008	−0.588	−0.750 to −0.359	<0.0001
$R50_{n}$	−0.406	−0.623 to −0.131	0.005	−0.528	−0.710 to −0.281	0.0002
$R90_{n}$	0.088	−0.207 to 0.369	0.559	−0.026	−0.314 to 0.266	0.863

**Table 11:** Correlations between changes in compactness-related metrics (ΔMetric) and changes in the total Sharp/van der Heijde score (ΔTSS) (n = 46).

ΔMetric	r	95% CI	*p*
ΔOSS	0.056	−0.238 to 0.340	0.713
ΔOSSD	−0.170	−0.439 to 0.127	0.259
ΔRMSR	−0.367	−0.594 to −0.086	0.012
ΔMPD	−0.352	−0.583 to −0.069	0.016
ΔR50	−0.168	−0.437 to 0.129	0.265
ΔR90	−0.335	−0.570 to −0.050	0.023
$\Delta RMSR_{n}$	−0.048	−0.334 to 0.246	0.751
$\Delta MPD_{n}$	−0.068	−0.351 to 0.227	0.653
$\Delta R50_{n}$	0.061	−0.224 to 0.345	0.689
$\Delta R90_{n}$	0.147	−0.150 to 0.419	0.331

## Data Availability

The data presented in this study are available on request from the corresponding author, as the legal procedures regarding permission for public data sharing at the institution where the data were generated have not yet been completed; the data will be made publicly available once these procedures are finalized.

## Supplementary Materials

The following supporting information can be downloaded at: https://www.mdpi.com/article/10.3390/jimaging12020087/s1, Figure S1: Correlations between centroid-based compactness metrics and joint space narrowing (JSN) in the entire cohort. Figure S2: Correlations between the wrist joint space narrowing subscore of the Sharp/van der Heijde score (JSNS) and compactness-related metrics at baseline (BL) for the combined left and right hands. Figure S3: Correlations between the wrist joint space narrowing subscore of the Sharp/van der Heijde score (JSNS) and compactness-related metrics at follow-up (FU) for the combined left and right hands. Figure S4: Correlations between bilateral summed wrist joint space narrowing subscores of the Sharp/van der Heijde score (JSNS) and all evaluated compactness-related metrics at baseline (BL). Figure S5: Correlations between bilateral summed wrist joint space narrowing subscores of the Sharp/van der Heijde score (JSNS) and all evaluated compactness-related metrics at follow-up (FU). Figure S6: Correlations between centroid-based compactness metrics and the wrist joint space narrowing subscore of the Sharp/van der Heijde score (JSNS) in the left hand at baseline (BL). Figure S7: Correlations between centroid-based compactness metrics and the wrist joint space narrowing subscore of the Sharp/van der Heijde score (JSNS) in the left hand at follow-up (FU). Figure S8: Correlations between centroid-based compactness metrics and the wrist joint space narrowing subscore of the Sharp/van der Heijde score (JSNS) in the right hand at baseline (BL). Figure S9: Correlations between centroid-based compactness metrics and the wrist joint space narrowing subscore of the Sharp/van der Heijde score (JSNS) in the right hand at follow-up (FU). Figure S10: Correlations between centroid-based compactness metrics and the wrist joint space narrowing subscore of the Sharp/van der Heijde score (JSNS) in the left hand at baseline (BL) and follow-up (FU). Figure S11: Correlations between centroid-based compactness metrics and the wrist joint space narrowing subscore of the Sharp/van der Heijde score (JSNS) in the right hand at baseline (BL) and follow-up (FU). Figure S12: Correlations between centroid-based compactness metrics and the total Sharp/van der Heijde score (TSS) in the entire cohort. Figure S13: Correlations between centroid-based compactness metrics and the total Sharp/van der Heijde score (TSS) at baseline (BL). Figure S14: Correlations between centroid-based compactness metrics and the total Sharp/van der Heijde score (TSS) at follow-up (FU).
