# Supplementary material for: Automated Compactness Quantitative Metrics for Wrist Bone on Conventional Radiography in Rheumatoid Arthritis: A Clinical Evaluation Study
Source: J Imaging. 2026 Feb 18;12(2):87. doi: 10.3390/jimaging12020087 (PMC12941718; doi:10.3390/jimaging12020087)
Supplement: Supplementary file 1 [file jimaging-12-00087-s001.zip › AP_DPM_Evaluation Supplementary File.pdf]

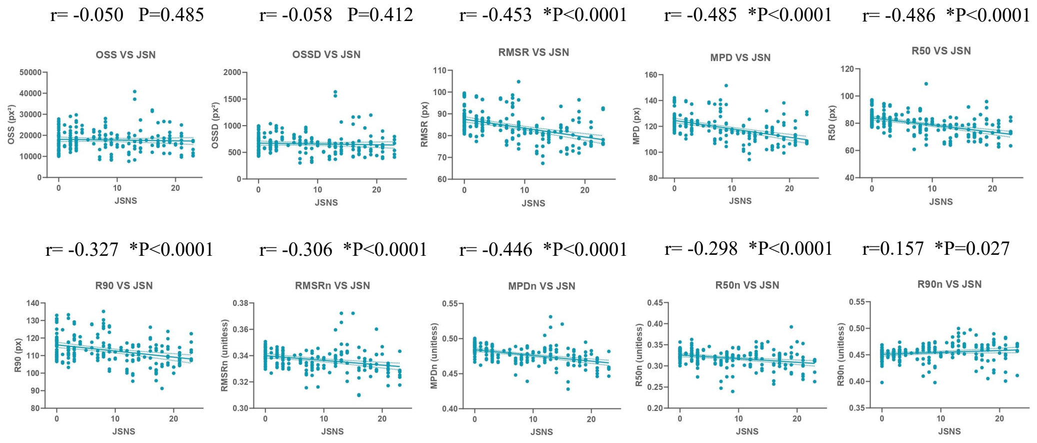

Figure S1. Correlations between centroid-based compactness metrics and joint space narrowing (JSN) in the entire cohort.

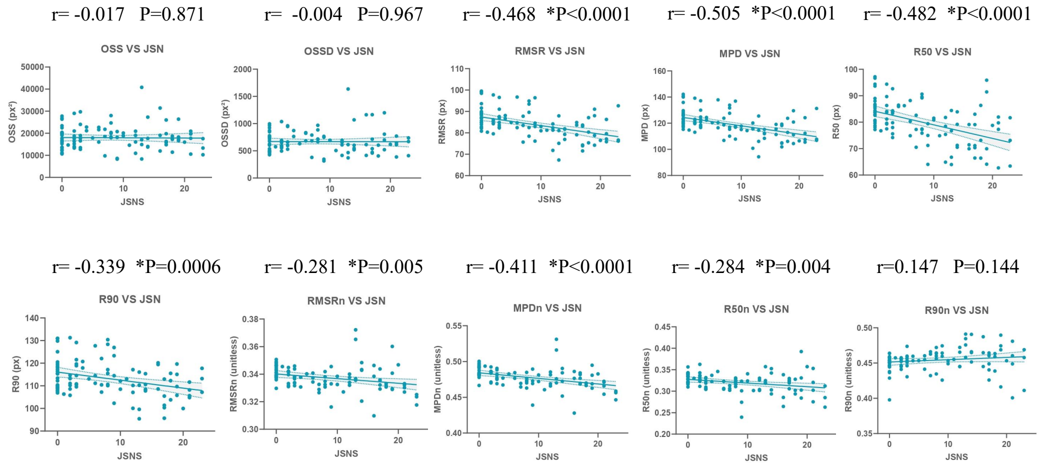

Figure S2. Correlations between the wrist joint space narrowing subscore of the Sharp/van der Heijde score (JSNS) and compactness-related metrics at baseline (BL) for the combined left and right hands.

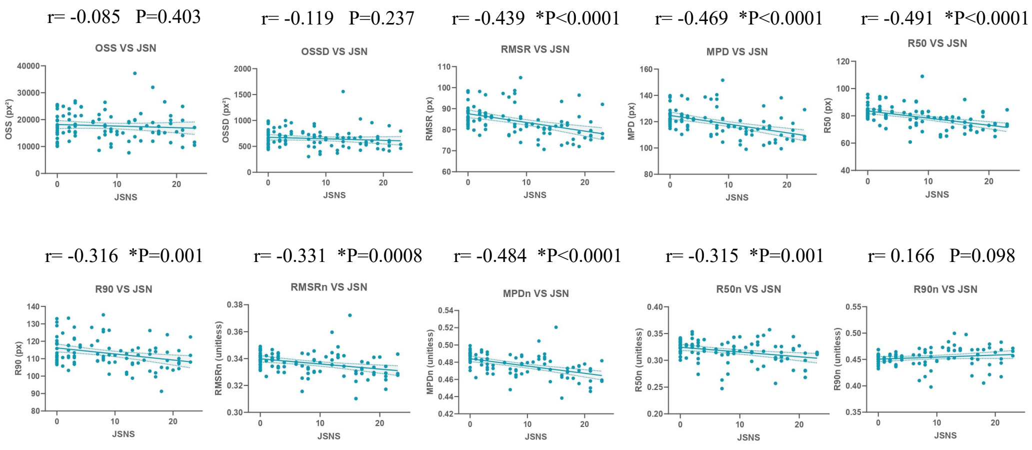

Figure S3. Correlations between the wrist joint space narrowing subscore of the Sharp/van der Heijde score (JSNS) and compactness-related metrics at follow-up (FU) for the combined left and right hands.

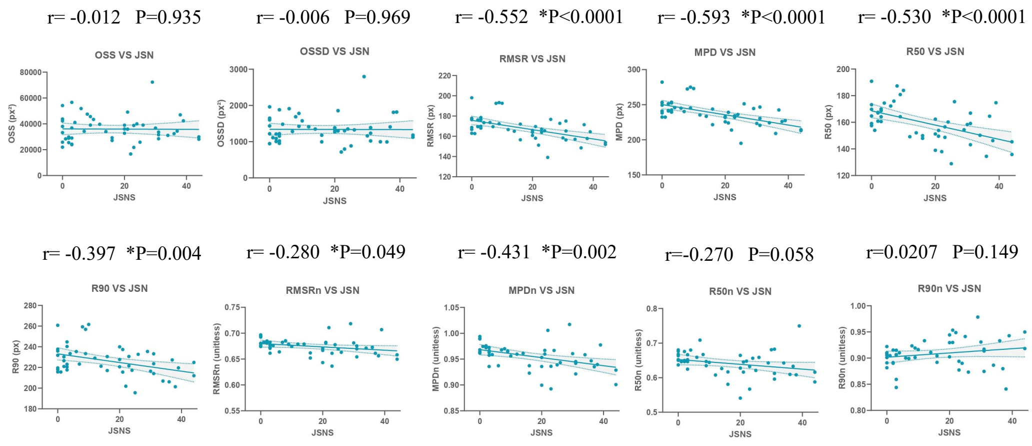

Figure S4. Correlations between bilateral summed wrist joint space narrowing subscores of the Sharp/van der Heijde score (JSNS) and all evaluated compactness-related metrics at baseline (BL).

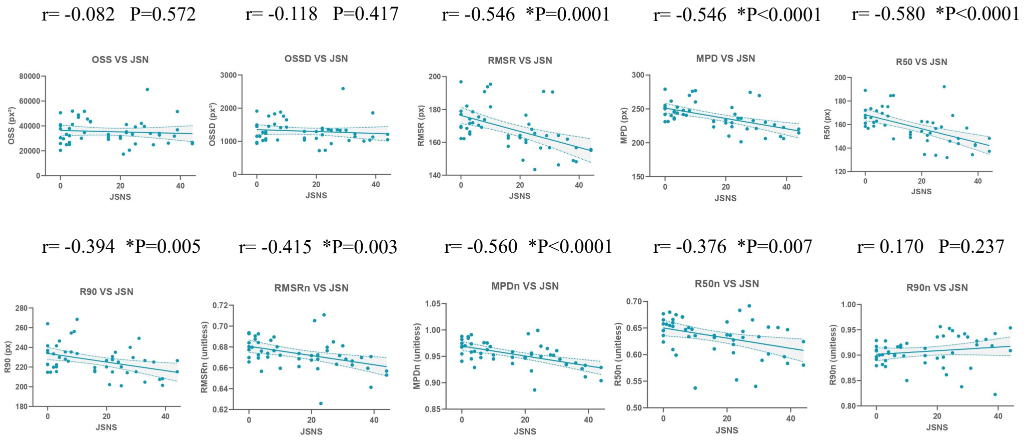

Figure S5. Correlations between bilateral summed wrist joint space narrowing subscores of the Sharp/van der Heijde score (JSNS) and all evaluated compactness-related metrics at follow-up (FU).

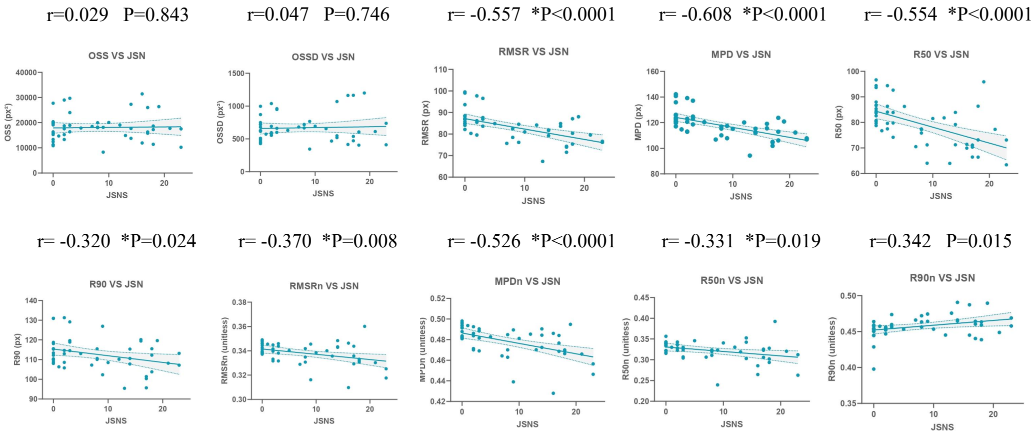

Figure S6. Correlations between centroid-based compactness metrics and the wrist joint space narrowing subscore of the Sharp/van der Heijde score (JSNS) in the left hand at baseline (BL).

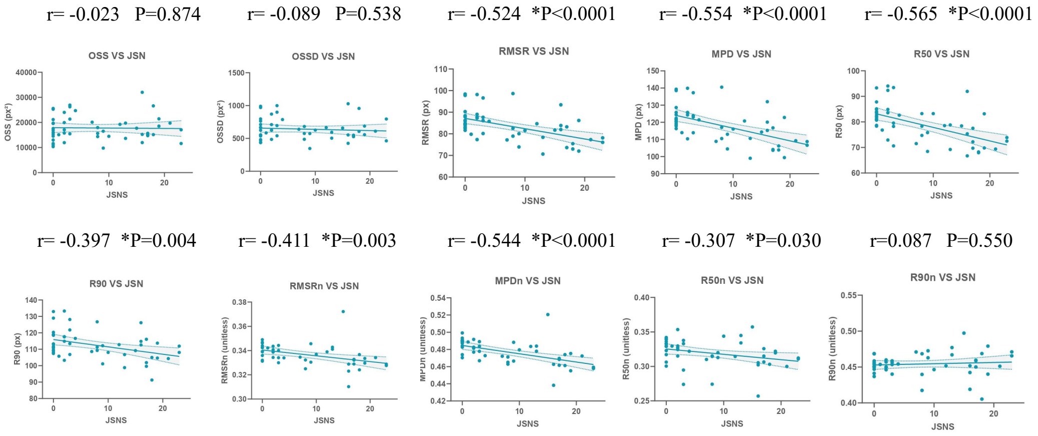

Figure S7. Correlations between centroid-based compactness metrics and the wrist joint space narrowing subscore of the Sharp/van der Heijde score (JSNS) in the left hand at follow-up (FU).

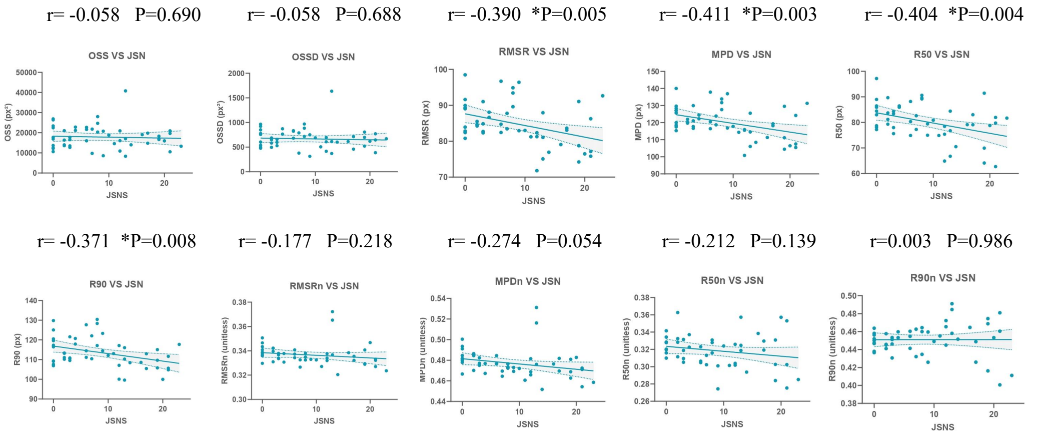

Figure S8. Correlations between centroid-based compactness metrics and the wrist joint space narrowing subscore of the Sharp/van der Heijde score (JSNS) in the right hand at baseline (BL).

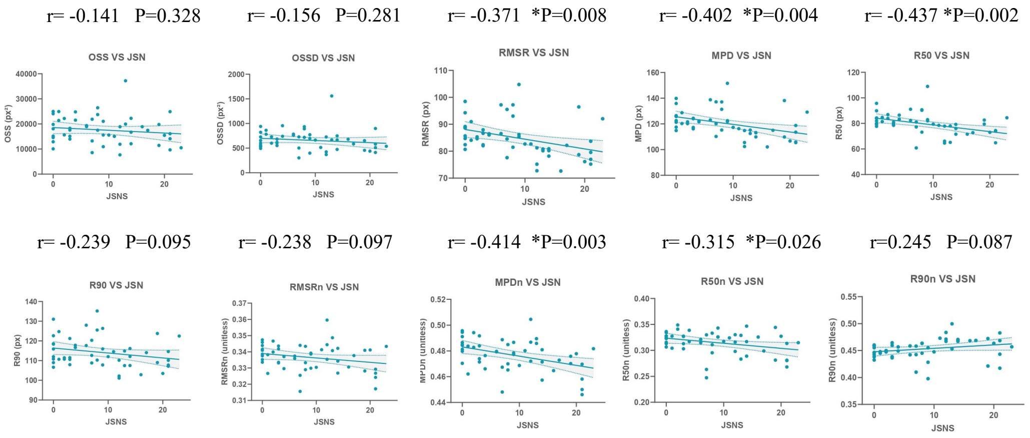

Figure S9. Correlations between centroid-based compactness metrics and the wrist joint space narrowing subscore of the Sharp/van der Heijde score (JSNS) in the right hand at follow-up (FU).

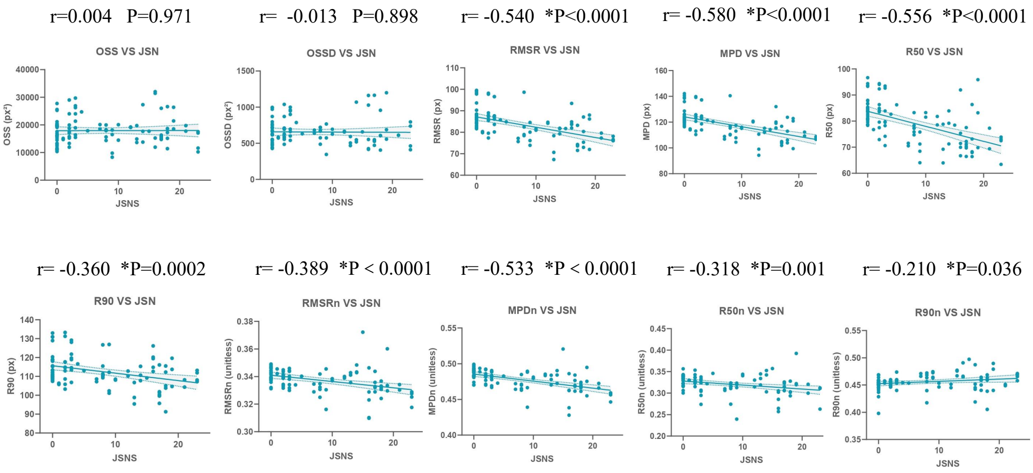

Figure S10. Correlations between centroid-based compactness metrics and the wrist joint space narrowing subscore of the Sharp/van der Heijde score (JSNS) in the left hand at baseline (BL) and follow-up (FU).

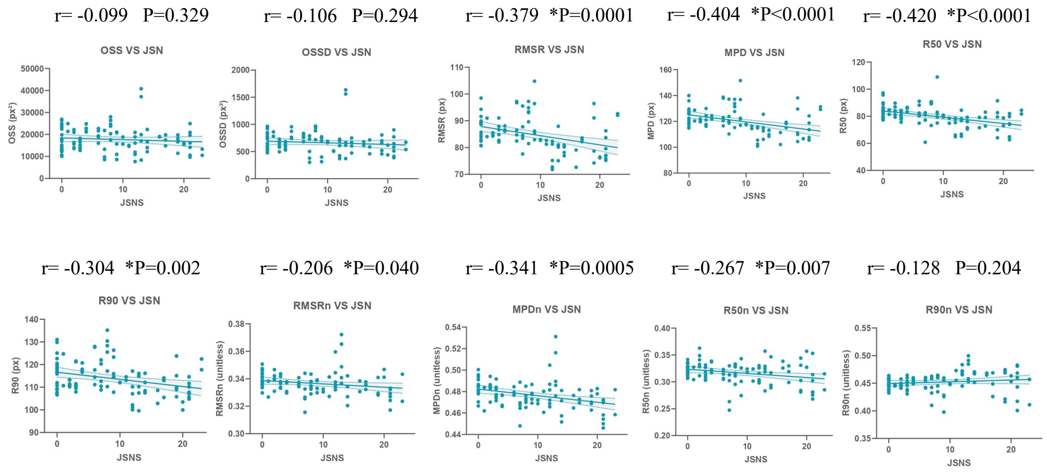

Figure S11. Correlations between centroid-based compactness metrics and the wrist joint space narrowing subscore of the Sharp/van der Heijde score (JSNS) in the right hand at baseline (BL) and follow-up (FU).

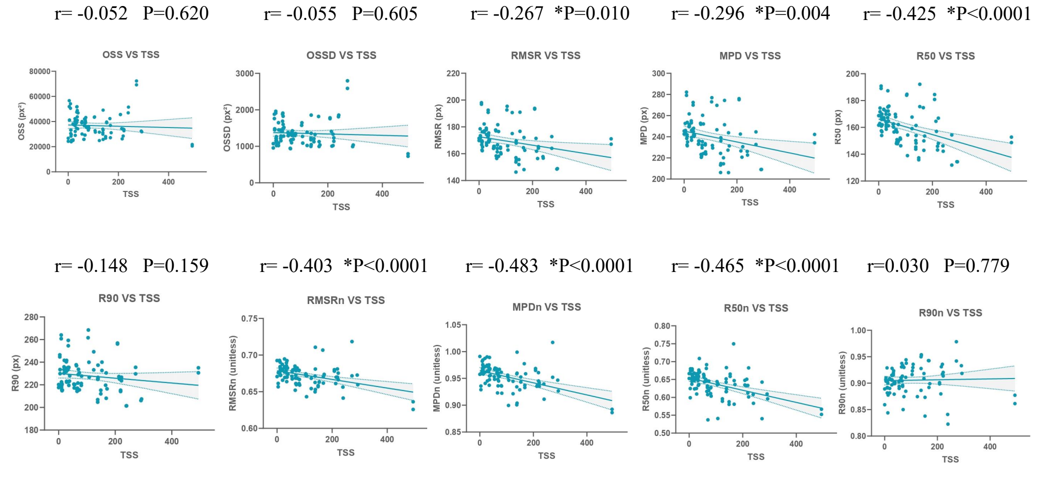

Figure S12. Correlations between centroid-based compactness metrics and the total Sharp/van der Heijde score (TSS) in the entire cohort.

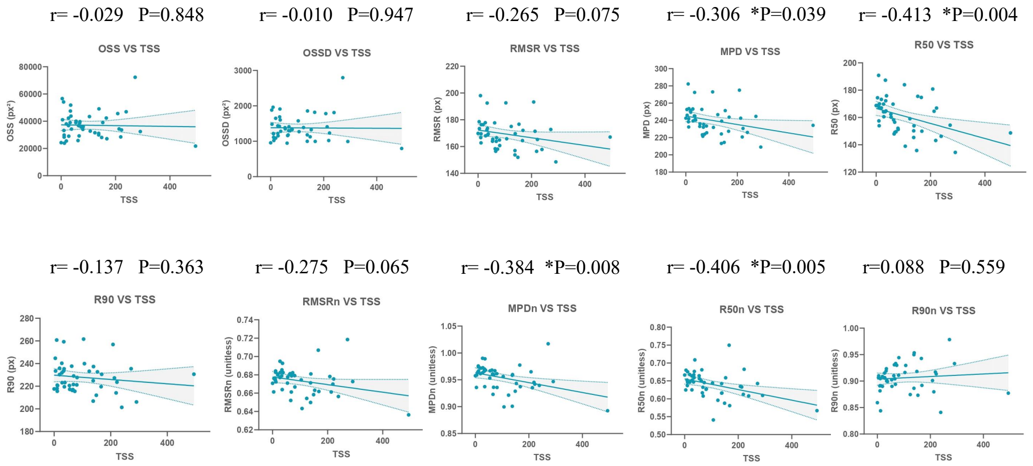

Figure S13. Correlations between centroid-based compactness metrics and the total Sharp/van der Heijde score (TSS) at baseline (BL).

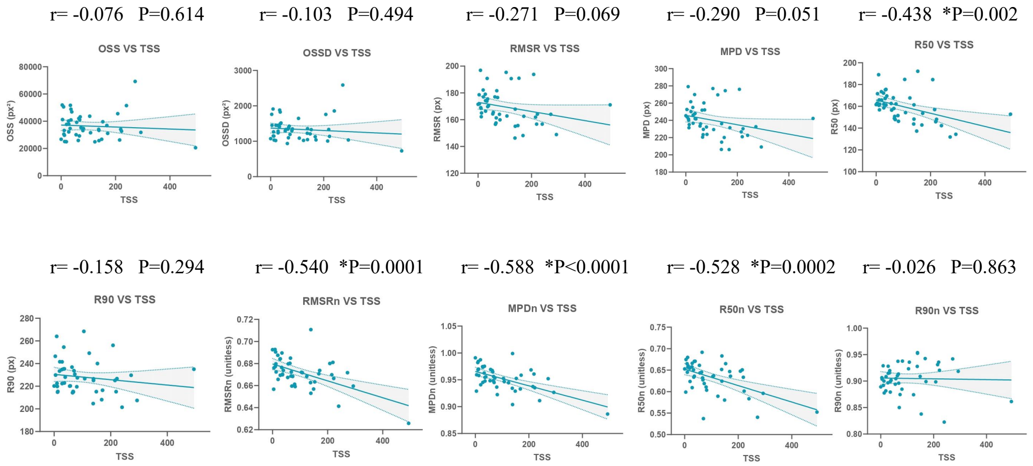

Figure S14. Correlations between centroid-based compactness metrics and the total Sharp/van der Heijde score (TSS) at follow-up (FU).
